# Operando Raman Shift Replaces Current in Electrochemical Analysis of Li-ion Batteries: A Comparative Study

**DOI:** 10.3390/molecules26154667

**Published:** 2021-08-01

**Authors:** Mariusz Radtke, Christian Hess

**Affiliations:** Eduard-Zintl-Institute, Physical and Surface Chemistry of Nanomaterials, Technical University of Darmstadt, Alarich-Weiss Str. 8, 64287 Darmstadt, Germany; christian.hess@tu-darmstadt.de

**Keywords:** lithium-ion battery, Butler–Volmer kinetics, operando Raman spectroscopy, kinetics

## Abstract

Li-rich and catalytically active γ-$Li_{x}V_{2}O_{5}$ (x = 1.48) was investigated as a cathode for its heterogeneous charge transfer kinetics. Using a specially designed two-electrode system lithium half cell, Butler–Volmer analysis was performed, and Raman spectra were acquired in 18 mV intervals. A direct correlation was observed between the Raman shift of the active modes $A_{g}$,$B_{g}$, $A_{u}$, and $B_{u}$, and the development of the Faraday current at the working electrode. The Raman intensity and the Raman shift were implemented to replace the current in a Tafel plot used for the analysis of Butler–Volmer kinetics. Striking similarities in the charge transfer proportionality constants α were found for current and Raman-based analysis. The potential of this new method of Raman-aided electrochemical detection at the diffraction limit is discussed.

## 1. Introduction

Energy storage devices in form of lithium-ion batteries are ubiquitous in everyday applications, which span from the use of externally charged gadgets to even backup systems of the large power grids [1]. While the generation of electricity is no longer a challenge, its conservation still poses a serious bottleneck, which is often caused by energy overproduction [2]. Among the batteries, there are many different energy storage systems available on the market for use in the industry today, which are building blocks of power grids and driving forces allowing energy consumption [3]. The Ragone plot compares energy density with power density and allows researchers to estimate what kind of storage device (battery, capacitor, or a hybrid) is appropriate for which type of application, and whether it is suitable for a large industrial system or rather for a notebook-sized device [4]. The devices and systems are being perfected in ongoing research processes; nevertheless, the exact mechanism of operation of, for example, heterogeneous lithium-ion batteries are still unknown on the microscopic and atomic scale [5].

While the macroscopic implementation of energy storage systems is well utilized, the microscopic mechanism underlying the reposition phenomenon is still under question [6]. In the case of complex nickel–manganese–cobalt-oxide-layered materials (NMCs), the lithium migration is responsible for charge buildup and increase in energy density, in addition to having other effects including carbon reorganization or buildup of a solid–electrolyte interphase (SEI) [7]. The reduction–oxidation (RedOx) induced movement of other cathode components, even the carbon movements are theorized to play a crucial role in the operation of a lithium-ion battery (LIB) [8].

Many simulations attempted to fully elucidate the structural changes occurring at the cathode and other parts of a battery during ongoing charge/discharge cycles, which led to a flourishing of the field of operando investigations of batteries [9,10,11]. A plethora of systems have been developed in order to spectroscopically investigate the structural and also vibrational/electronic changes occurring at the cathode. Prominent examples among these are operando X-ray diffraction (XRD) for potential-dependent structural changes within crystals, Raman spectroscopy for long- and short-range structural information, and X-ray photoelectron spectroscopy (XPS) or even sophisticated synchrotron-source-extended X-ray absorption fine structure (EXAFS) for changes in the electronic structure [12,13,14,15,16].

In order to have a deeper insight into the mechanism of battery operation, the distinction between electrochemical processes of charge transfer and mass transport is needed for the disambiguation of the merits governing the kinetics of chemical and physical processes [17]. As heterogeneous charge transfer and mass transport are two main phenomena occurring within the battery, the kinetic parameters of both should be elucidated by the use of dedicated electrochemical techniques spanning from polarization (Tafel plot) to electrochemical impedance spectroscopy (EIS) [18].

For electrochemical techniques, the signal readout is spatially restricted due to the size of a working electrode. In the case of electrodes commonly used in research, with the exclusion of atomic force microscopy (AFM)-based approaches with cantilever tips reaching single-atom resolution, the electrochemical output is a sum of the superimposed signals originating from the areas reaching several hundreds of square micrometers. On the other hand, joined spectroelectrochemical operando methods, e.g., based on Raman spectroscopy, allow for the understanding of the changes limited in resolution only by the size of the laser spot. Raman spectra, therefore, yield a point measurement, whereas the electrochemical scan represents an integral approach.

By careful adaptation of an integral electrochemical technique and an optical point method such as Raman spectroscopy, it is possible to gain additional insight into changes occurring in each building block of the cathode (active material, binder, and conductivity enhancers) at the given potential [19]. By the analysis of Raman active modes acquired at a given polarization potential, e.g., on the cathode, one may even perform mode-sensitive Raman spectroelectrochemistry.

As Raman spectroscopy shows vibrational modes of the active material, electrolyte, binder, and conductive carbon, it allows researchers to even elucidate whether the lithium migration occurs in a specific pathway involving well-defined geometry influencing Raman bands. The properties of the Raman bands and their intensity are susceptible to the adsorption of metallic species on the working electrode, which foremost causes differences in optical skin depth and reflection of the laser rather than its scattering. Electrochemistry, on the other hand, provides the integral kinetic characterization of the process [20]. In the case of, e.g., $Li_{x}V_{2}O_{5}$, the potential dependence of the V=O symmetric stretch can be used to elucidate the state of lithiation of the lithium vanadium pentoxide [21].

As the chemical reactions are the driving force of energy storage in Li-ion battery, the kinetics of charge transfer in electrochemically reversible Red-Ox reactions are most commonly investigated by the Butler–Volmer and Tafel models [22]. The drawback of a pure electrochemical approach toward the elucidation of charge transfer merits is the integral character unless electrochemical microscopic approaches are undertaken. Raman spectroscopy aids in the detection of changes in the vibrational structure of the cathode material via analysis of phonons. The disadvantage is the incompatibility of the integral electrochemical method utilizing the whole area of the cathode and point character of the Raman measurement with a laser spot size of several micrometers. This disadvantage can be resolved, for example, by coupling electrochemical AFM with Raman spectroscopy. The cantilever would then be used as a quasi-atomic-sized electrochemical working electrode, which was found to resolve electrochemistry in sub-nm lateral scale [23]. As Raman spectroscopy is a diffraction-limited technique displaying Abbé characteristics, it can only display resolution similar to AFM by advanced techniques of stimulated emission depletion (STED) or tip-enhanced Raman spectroscopy (TERS) [23,24]. Those combinations are, nevertheless, outside the scope of this study; in our work, we rather highlight the use of complementary techniques of various resolutions.

This study shows the possibility of using a laser signal instead of a current readout during the electrochemical scan, therefore allowing the spatial resolution of processes occurring within the battery to be only limited by the light diffraction and the Raman cross section of the given assembly of molecules [25]. A remarkable similarity between the results calculated from the standard and integral electrochemical tests and those of the Raman-mode-dependent spectroelectrochemistry is presented by an example of a Tafel analysis. This study introduces a new tool for elucidating mechanisms occurring within the battery with higher spatial precision based on the statistical distribution of Raman-active modes at the diffraction limit.

Lithium vanadium pentoxide was chosen as the material due to its relatively large Raman cross section and well-established spectral properties. It has allowed a reliable system to be generated that exhibits a well-defined spectroelectrochemical response upon polarization.

## 2. Results and Discussion

**Figure 1:**
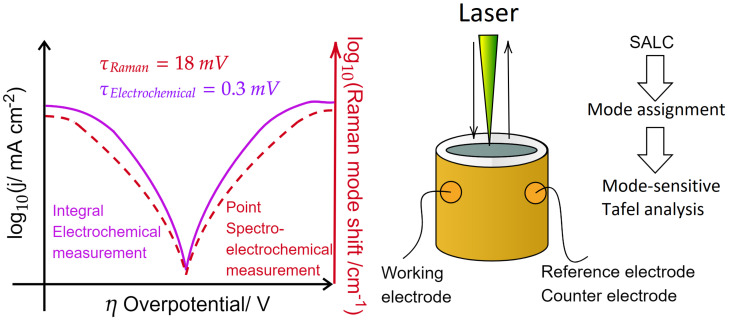
Principle of the mode-sensitive Raman spectroelectrochemical measurement. A laser (in confocal setup) shines on a two-electrode cell, equipped with borosilicate window shielding it from the ambient atmosphere. The Raman signal is acquired every 18 mV, and the electrochemical signal is at a rate of 0.3 mV/s. Comparison of the electrochemical signal and shift of the Raman mode in logarithmic scale allows us to monitor the performance of charge transfer kinetics according to the Butler–Volmer equation via Tafel plot analysis.

The operando Raman spectroelectrochemical analysis of the charge transfer occurring within the Li-ion battery was performed in a specially designed electrochemical cell supplied with borosilicate glass, allowing the laser to freely penetrate into the interior of the cathode. Such an approach allows correlating every Raman spectrum to a specific potential, as outlined in Figure 1. The delay caused by the time necessary for the acquisition of each Raman spectrum simultaneously with the constantly ongoing electrochemical Tafel analysis was set to 240 s. This time span was needed in order to collect the Raman spectra with an acceptable signal-to-noise ratio. The Raman spectrum was, therefore, recorded within a span of 18 mV, while the electrochemical scanning rate was set as 0.3 mV/s. In a potential span of 2–5.2V vs. $Li/Li^{+}$ the measurement took about 7 h to complete, and the assumption of a nonchanging steady-state of the fresh cathode was made.

**Figure 2:**
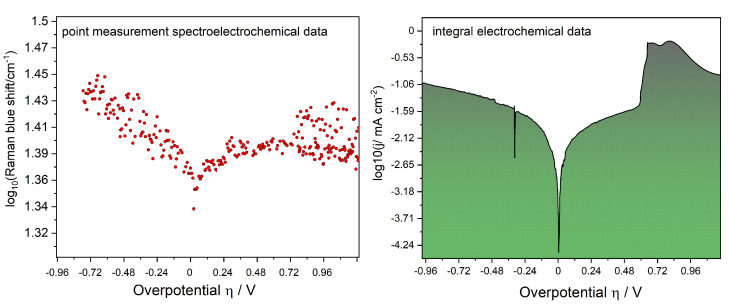
Comparison of the integral Tafel plot in a two-electrode system with γ-$Li_{x}V_{2}O_{5}$/carbon black as a cathode, compared to the 3 µm Raman spectroelectrochemical Tafel plot (with the blue shift defined as: $\Delta \nu =\nu_{U(0V,0s)}-\nu_{U(V,t)}$). A comparison of the results is provided in Table 1. Note the influence of the polarization on the steep slope of the cathodic part and resulting proportionality constants in Table 1, and the fact that the blue shift upon reverse polarization is seen as a redshift. The position of the B1g active mode is 901 cm${}^{-1}$ (at OCP, with no polarization).

The intensity of the Raman measurement was found to be dependent on the applied potential. The laser readout was found to follow the trends of the current developed at the cathode. This observation has laid the base for the whole analysis and concept within this study. As shown in Figure 2, the trends were found to be similar to such an extent that the typical Tafel analysis of charge transfer was performed by replacing the current density with the shift (Δν) of the Raman mode of γ-$Li_{x}V_{2}O_{5}$. The results of this analysis were gathered in Table 1.

As the intensity of the Raman measurement was found to be highly dependent on the applied potential, so were also the band characteristics in form of the full width at half maximum and the position of the global maximum position. The laser readout was found to follow the trends of the current developed at the cathode. This observation has laid the base for the whole analysis and concept within this study. As shown in Figure 2, the trends were found to be similar to such an extent that the typical Tafel analysis of charge transfer was performed by replacing the current density with the shift (ν) of the Raman mode of γ-$Li_{x}V_{2}O_{5}$. The results of this analysis were gathered in Table 1, whereby an example of the full calculation pathway of the heterogeneous charge transfer constant is provided in the Supporting Information Section (see Supporting Information, Figure S5).

The difference between the electrochemical and spectroelectrochemical measurements was found to be mode sensitive. The electrochemical response involved the whole material under investigation, while the spectroscopic signal did not follow the applied potential in the same fashion for all the Raman active modes. A more detailed discussion of this observation will be provided in Section 2.3. The electrochemical measurement was integral in its character, and the current readout involved the signal development at the whole cathode. In the spectroelectrochemical measurement, the current readout was replaced by the Raman signal, which followed the applied potential in form of a red- or blueshift (based on the position of the global maximum after mathematical fitting). Depending on the position of the polarization, the blueshift in Figure 2 is represented by the rise of the $log_{10}\Delta \nu$(cm${}^{-1}$) values, and the redshift by their dropping, respectively. The blueshift represents the oxidation caused by the de-lithiation, and the redshift, by the reduction of the active material.

### 2.1. Electrochemical Measurements on γ-$Li_{x}V_{2}O_{5}$

As a model of the measurement of charge transfer, a Tafel plot was chosen due to the linear polarization character allowing us to precisely estimate the kinetic merits. The Tafel plot and respective fitting of the cathodic and anodic slopes were performed according to the Butler–Volmer equation, Equation (1) as follows:

(1) $$j_{\mathrm{Ox}}=\frac{i}{nFA}=c_{\mathrm{Ox}}\left(0,t\right)\;\cdot \;k^{0}\;\cdot \;e^{\left[-\alpha \frac{nF}{RT}\left(E-E^{0}\right)\right]}-c_{\mathrm{Red}}\left(0,t\right)\;\cdot \;k^{0}\;\cdot \;e^{\left[\left(1-\alpha \right)\frac{nF}{RT}\left(E-E^{0}\right)\right]}$$

where *j* is the current density, *i* the current, *n* the number of transferred electrons during the heterogeneous charge transfer reaction (assumed as 1 in this study), *F* the Faraday constant, *A* the surface of the electrode, $c_{Ox}$ the concentration of the electrochemically oxidized species in the cathodic part, $c_{Red}$ the concentration of electrochemically reduced species in the anodic part of the Tafel plot (see Figure 2), $k^{0}$ the standard reaction constant, and α the charge transfer coefficient (proportionality factor).

As the standard form of the Butler–Volmer equation may be reduced to the Tafel equation, only the proportionality constants were calculated (see Table 1). In the case of the cathodic part of the Tafel plot, α was found to be 0.13 from the overpotential in the linear region between 410 and 460 mV, while the cathodic slope and its analysis yielded the proportionality factor of 0.87. This indicates that the oxidation reaction is prevalent in the case of γ-$Li_{x}V_{2}O_{5}$, which are integral results arising from the whole cathode. Similar results were obtained by purely electrochemical analysis, where the cathodic charge transfer coefficient (proportionality factor in this study) was also estimated as 0.13 [26].

The variation of the charge transfer coefficient respective to the Raman active mode indicates which bonds are affected the most by the abstraction of lithium from the $Li_{x}V_{2}O_{5}$ structure. Due to the interaction of Li${}^{+}$ with V=O ($A_{g}$, V=O, “1” in Table 1), the vanadyl stretch V=O exhibits the most drastic changes ($\alpha_{cathodic}$ = 0.88), and the resulting change in electronic structure perpetuates the neighboring V-O(“2” in Table 1) bond ($\alpha_{cathodic}$ = 0.90). $B_{2g}$ (V-O “3” in Table 1) and $B_{3g}$ (V-O, 4 in Table 1) modes are slightly less affected by this perturbation, and the $B_{u}$ mode, the presence of which is currently not understood, appears to be a mixture of all the affected modes and exhibits the highest perturbation ($\alpha_{cathodic}$ = 0.92). This effect might be caused by the electronic perturbation arising from Li${}^{+}$ abstraction, which translates to the more pronounced behavior in the Raman spectrum (the $B_{u}$ mode should be silent or weak due to the acoustic properties) [27,28].

### 2.2. Raman Measurements on γ-$Li_{x}V_{2}O_{5}$

The Raman-active modes of γ-$Li_{x}V_{2}O_{5}$ were obtained by applying the symmetry-adapted linear combinations (SALCs), according to Popovic et al. [28].

(2) $$\Gamma =16A_{g}+8B_{g}+8A_{u}+16B_{u}$$

Not all theoretically predictable Raman-active modes could be found in our room-temperature spectra, in contrast to the studies by Popovic et. al., conducted at cryogenic temperatures [28]. The de-intercalation of lithium manifested itself in form of a blueshift, which has been related to an enhancement of the crystallinity of the material under investigation [29].

**Figure 3:**
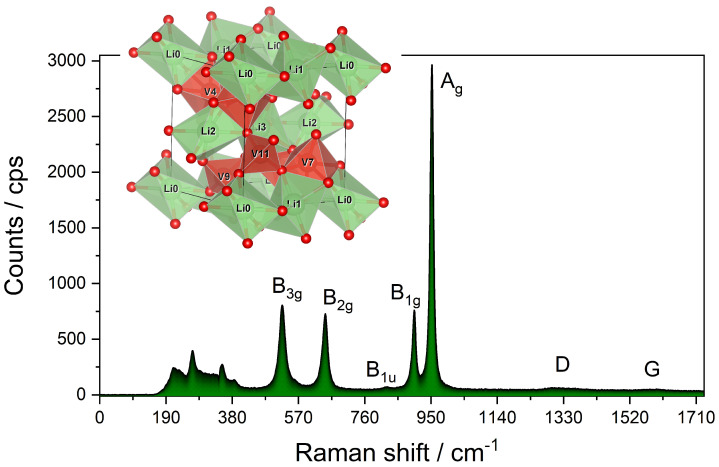
Raman spectrum and symmetry adapted linear combination of the active material. The spectrum shows the signals of carbon black (D and G bands) co-mixed in the cathode in this study.

As Li${}^{+}$ is being detached from the crystallographic unit cell of $Li_{x}V_{2}O_{5}$, the stress-levels connected with a drop in crystallinity caused by Li accommodation between $V_{2}O_{5}$ sheets, and the lattice phonons directly correlated with increasing the elastic constant of the crystal (due to the increased presence of lithium-poor $Li_{x-n}V_{2}O_{5}$ + nLi${}^{+}$, exhibiting more $V_{2}O_{5}$ behavior) become more pronounced [29,30] Due to the deintercallation of Li${}^{+}$, the increased elastic constant (less stress posed on a crystal) will, therefore, result in a blueshift. The highly lithiated γ-$Li_{x}V_{2}O_{5}$, with a V=O stretch at 953 cm${}^{-1}$, undergoes polymorphic transformation caused by oxidative de-lithiation to pure $V_{2}O_{5}$ with a V=O stretching vibration at 990 cm${}^{-1}$ (see Supporting Information, Figures S1 and S4). Figure 3 shows the respective Raman spectrum with band assignments, which was used in the spectroelectrochemical analysis of the experimental results in the following subchapter. The Raman-active modes chosen in the analysis were based on the clear visibility in the electrochemical scan, as part of the spectrum was blurred by the presence of the electrolyte LP30. The Raman modes in the range 150–400 cm${}^{-1}$ correspond to polymerized -O-V-O- vibrations [31].

As explained by Badour et al., polymorphic changes within $Li_{x}V_{2}O_{5}$ occur due to the various amount of coordinated lithium between the $V_{2}O_{5}$ layers [32] and can be followed by Raman spectroscopy. As the structure of pure $V_{2}O_{5}$ is being altered by accommodation of lithium between the layered structure of $V_{2}O_{5}$ sheets, the V=O bond is weakened as a cause of Li${}^{+}$ coordination and shows a redshift from 990 cm${}^{-1}$ in pure $V_{2}O_{5}$ to 953 cm${}^{-1}$. The V=O symmetrical stretch ($A_{g}$ mode) of the lithium-rich γ-polymorph alters in the course of the electrochemical oxidation. The material starts losing lithium and the pure behavior of $V_{2}O_{5}$, nondistorted by coordination, becomes more pronounced [27].

The presence of coordinated Li on the vanadyl V=O may be the source of variation in Raman intensity. This may be dictated by the fact that electrochemically reduced lithium may have an influence on the optical skin depth of the laser. Accumulated near vanadium pentoxide, lithium will not be Raman active in its metallic state but may still alter the properties of the V=O band. The elemental lithium may reflect the laser instead of scattering it; therefore, the intensity of the Raman spectrum may rise, as may the full width at half maximum of the V=O band (A1g mode), as the heterogeneity of the electrode is increased; however, in the experiment, the intensity was not observed to change. Only those modes responsible for the interaction with vanadyl V=O are expected to be altered by the applied potential. The higher the lithium content is, the more pronounced redshift the $A_{g}$ mode is expected, which was confirmed by the results in this study. Additionally, the behavior of the V=O bond and its sideband (especially $B_{1g}$ mode) follow the same trend induced by stepwise de-lithiation of $Li_{x}V_{2}O_{5}$ [33].

The Raman probing depth, as an interplay between bulk properties of influenced and surface species, was already tackled by others [34]. In our case, the penetration depth poses a challenge, which was investigated by a z-scan, and the good comparison of electrochemical merits (integral technique) with the laser-point methodology is to be referred to the sample‘s homogeneity and homogeneous charging. The representative z-scan on a standard ((400) single crystal CVD-grown diamond) can be seen in Figure S10, Supporting Information. We note that the data presented in this study are acquired in Raman focus, and the depth-profile investigation is currently a matter of our separate study on multidimensional Raman spectroelectrochemistry.

By translation of group theory into the chemical environment, as $Li_{x}V_{2}O_{5}$ is composed of the main V=O (V=O(1)) stretch ($A_{g}$ mode), the following $B_{1g}$ (V-O(2)), $B_{2g}$ (V-O(3)) and $B_{3g}$ (V-O(4)) modes arise from stretching vibrations of V-O bonds with the corresponding oxygen atoms within the structure. This mode, therefore, would arise from the V-O stretching vibration within the crystallographic unit. We acknowledge the presence of the very weak mode at 842 cm${}^{-1}$, which most probably occurs due to the silent activity and will be more pronounced in the IR spectrum, as was speculated by others [28]. This mode was described as IR LO mode, which we speculate to be a partially acoustic mode ($B_{1u}$, IR active/Raman silent). As mentioned by Popovic et al., the presence of this mode is not fully understood. Table 1 contains both the description of chemical bonds and corresponding irreducible representations.

The observation of a blueshift during oxidation and a redshift upon reduction is in good agreement with the observation of a higher proportion of the oxidation reaction by purely electrochemical analysis of the Tafel plot of the whole cathode and spectroelectrochemical analysis for the $A_{g}$, $B_{1g}$, $B_{1u}$, $B_{2g}$, and $B_{3g}$ modes. It might be referred to as the compensation of the positive charge generated by oxidation in the active material. In the case of linear potential sweep characterization of $Li_{x}V_{2}O_{5}$, a similar behavior was indicated by others [32,35]. As the strain within the $Li_{x}V_{2}O_{5}$ was lowered, it impacted the further electrochemical and spectral behavior. Figure 3 shows the respective Raman spectrum with band assignments, which was used in the spectroelectrochemical analysis of the experimental results in the following subchapter. The Raman-active modes chosen in the analysis were based on the clear visibility in the electrochemical scan, as part of the spectrum was blurred by the presence of the electrolyte LP30. The lack of binder did not impair the electrochemical characteristics of the oxide semiconducting material, and an exemplary differential pulse voltammogram (DPV) was gathered in the Supporting Information Section (see Supporting Information, Figure S2). To evaluate the lack of binder on conductivity characteristics, staircase-potential electrochemical impedance spectroscopy (SPEIS) was performed in the whole potential window with a 30 µV distance between each spectrum. Noticeably, no large increase in Warburg impedance was observed at higher potentials reaching 5.2 V vs. Li${}^{+}$/Li, which indicates neither the decomposition of the electrolyte nor the in situ generation of complexes with the cathode material occurred (see Supporting Information, Figure S2). To evaluate the current drop between each current probing in the Butler–Volmer analysis, a potentiostatic intermittent titration technique (PITT, see Supporting Information, Figure S6) was applied over the whole potential range, which indicated negligible decomposition of electrolyte above 4.8 V (see Supporting Information, Figure S2). The relaxation time was estimated to be 80 s, which is three times lower than the time needed for the recording of Raman spectra (240 s, which, with an acquisition rate of 0.3 mV s${}^{-1}$, corresponds to the 18 mV delay between the spectral Raman and electrochemical readout). This short time, therefore, confirmed the operando character of our study and requirements posed on the Butler–Volmer experiment.

### 2.3. Raman Spectroelectrochemical Measurements. Comparison of Electrical vs. Laser Readout

In order to transform the integral measurement to a diffraction-limited point scan, Raman spectroelectrochemistry was performed. The current readout in the Tafel plot was replaced by the shift of the respective Raman mode shift defined as follows:

(3) $$\Delta \nu =\nu_{U(0V,0s)}-\nu_{U(V,t)}$$

The assignment of the symbols in Equation (3) can be found in the caption of Figure 2. Briefly, $\nu_{U(0V,0s)}$ is the Raman shift due to the voltage (potential) change in time at 0 s (prior polarization), and $\nu_{U(V,t)}$ during the electrochemical experiment at the time ‘t’. This definition allows us to show the striking similarity between the electrochemical and spectroelectrochemical readouts in Figure 2. When only the Raman intensity was plotted as the *y*-axis of Figure 2, the Tafel plot was symmetrically mirrored along the *x*-axis.

In addition, not all Raman-active modes followed the trend of the Raman intensity following the applied potential, with $B_{3g}$ and $B_{1u}$ being the most prominent examples, and in the case of varying lithium content in $Li_{x}V_{2}O_{5}$, it was already covered by others [29]. Briefly, the vibrational modes of the electrochemically conductive materials can be altered by the presence of electric fields, which allows gaining insight into the charge transfer processes at the molecular level [36]. The static electric field generated during the electrochemical scan was found to have a direct influence on the position (Raman shift ν) and band characteristics of the Raman spectra, which applies to all modes. Only the Raman intensity (e${}^{-}$/s) was not uniform in response to all active modes. The Raman shift Δν in Equation (3) has nevertheless allowed for a representation enabling the calculation of kinetic merits in Table 1. The Raman shift was found to follow the trends arising from the development of the current at the cathode upon applying the potential in all vanadia-related modes of the active material presented in Figure 3, whereby the D and G bands from carbon black were not investigated here but will be part of a separate study. The peculiarity of the Raman spectroelectrochemical studies in $Li_{1.48}V_{2}O_{5}$ material (for stoichiometric considerations see Supporting Information, Figure S8), exhibiting considerably large blueshifts dictated by the polymorphizations (γ to ε), has resulted in the large logarithmic scale not crossing zero, unlike, for example, the $B_{1g}$ mode (see Supporting Infromation, Figure S5). Based on Equation (3), we were able to reach the 0 on the y-scale in all cases except the $A_{1g}$ mode shown in Figure 2. This mode referring to the V=O stretching vibration was highly affected by the Li${}^{+}$ migration. This discrepancy has nevertheless still allowed for the calculation of the merits needed to observe the similarity in the Butler–Volmer analysis. The $A_{1g}$ mode was chosen due to the representation of the V=O stretching vibration shortened or expanded by the movement of Li${}^{+}$.

The position of the Raman-active bands allowed the kinetic analysis to be performed in detail; nevertheless, it was not possible to do so by using only the intensity of the Raman bands (in e${}^{-}$/s) due to the lack of fluorescence suppression. In the case of using only the Raman shifts instead of Raman intensity, the small deviations between the electrochemical and spectroelectrochemical results in Table 1 speak in favor of applying mode-sensitive electrochemical Raman detection. The charge transfer in γ-$Li_{x}V_{2}O_{5}$ was found to be mostly governed by the anodic oxidation and de-lithiation, which is in good agreement with the shift of the, e.g., $B_{1g}$ mode presented in Figure 2. By the following observation of the redshift at higher potentials in Figure 2, the $V_{2}O_{5}$ sheets started to gain crystallinity as the charge generated during oxidation was being compensated, and the $Li_{x}V_{2}O_{5}$ started to undergo the phase transition from γ to ε polymorph [37,38]. The charge compensation and phase transformations were also manifested by a higher Raman intensity in the case of the $B_{1g}$ mode.

The minimum time needed for current linearization obtained from PITT measurements was the starting point for establishing the Raman measurement time in the dynamic region of the polarization. The relatively slow acquisition is dependent on both the Raman cross section of the material (the laser probing time can, therefore, be kept at a minimum in the case of $Li_{x}V_{2}O_{5}$, while the spectral resolution will increase with prolonged scanning time) but, moreover, on the time needed to reach the thermodynamic equilibrium after potential-induced perturbation. In order to accelerate the data acquisition needed for, e.g., Poisson statistics, further studies with much smaller potentials in nV and picoampere current regimes are needed (low-current measurement options).

## 3. Materials and Methods

### 3.1. Synthesis and Characterization of $\gamma -Li_{x}V_{2}O_{5}$

A total of 3.38 mmol of lithium carbonate (Sigma Aldrich, Taufkirchen, Germany, 99.9% purity trace metal basis) and 1.37 mmol of vanadium(IV,V) oxide (Alfa Asesar, Heysham, UK, 99.9% purity trace metal basis) were added to 60 mL of deionized water in two-neck flask charged with a magnetic stirrer and equipped with a reflux condenser and a pressure-regulated dropping funnel. The material was brought into reflux, and 3 mL of 20%-aqueous trisodium ascorbate trihydrate (Sigma Aldrich, Germany Reagent plus) was added into the refluxing mixture, which immediately turned pale yellow. The precipitate was collected by vacuum filtration, dried at 80 °C overnight, and annealed in an oven at 400 °C (heating rate 5 °C/min, annealing period 2 h and cooling rate 5 °C/min), yielding orange precipitate. The bright gold crystals were characterized by X-ray powder diffraction (XRD, Figure S7 in the Supporting Information) with a Cu K$\alpha_{1}$ X-ray source (STOE StadiP) and X-ray photoelectron spectroscopy (XPS) to confirm the material composition (Surface Science SX100, see Figure S9 in the Supporting Information). The wavelengths and sources of XRD was 1.54056 Å (Cu K α 1) and the XPS was Al K α source (9 kV, 10 mA). XPS spectra were treated in CASA 2.3.22PR1.0 software with Shirley’s baseline function. As XPS analysis revealed a value of x = 1.48, the $Li_{x}V_{2}O_{5}$ mentioned in the text refers to $Li_{1.48}V_{2}O_{5}$. Ex situ Raman spectra were collected over the sample and confirmed the presence of Li-V-O stretching vibrations and matched the literature for γ lithium vanadate. Raman spectra were recorded on Kaiser Optical Raman spectrometer equipped with Peltier-cooled-CCD camera and Cobolt 532 nm solid-state laser. The laser power was varied according to the measurement of interest. The ex situ measurements of $Li_{x}V_{2}O_{5}$ were recorded over a 60 s period of time at a power of 840 µW during the operando measurements. The transition of the γ to the ε-$Li_{x}V_{2}O_{5}$ phase was monitored by operando Raman spectroscopy (see Supporting Information, Figure S3). The electrode thickness was 0.48 mm, and the surface roughness was 600 nm, as established by AFM in tapping mode modus.

### 3.2. Butler–Volmer Measurements

All electrochemical measurements were performed with the help of a BioLogic VSP potentiostat (France). Tafel plot analysis was performed in the range of 2–5.2 V vs. Li/Li${}^{+}$ with a polarization scan rate of 0.3 mV/s and a relaxation time of 30 min. Raman measurements were performed on a Kaiser Optical Raman spectrometer equipped with a 532 nm green diode laser (Cobolt). The laser spot was estimated as 4.07 ± 0.52 µm, and the laser power was kept at 840 µW, yielding a fluence of 2.06 W/cm${}^{2}$. Raman spectra in the in situ/operando cell (EL-Cell GmbH, Hamburg, Germany) were acquired every 18 mV. The 0.3 inch-thick lithium foil was purchased from Alfa Aesar, The United Kingdom, LP30 electrolyte of battery grade was obtained from Sigma Aldrich, Germany. Electrochemical cells were assembled in an Ar-filled glove box with $O_{2}$ and $H_{2}O$ concentrations below 3 ppm. The separator used in this study was Whatman 10 µm filter paper. γ-$Li_{x}V_{2}O_{5}$ was synthesized according to a procedure described elsewhere [27]. The material under study was mixed in an 80:20 ratio with carbon black (battery grade, Sigma Aldrich, Germany) and pressed onto an $Al_{2}O_{3}$ grid under 3 tons of pressure. No binder was used in this study in order to preserve the purity of the output Raman signal. All Raman active bands were mathematically fitted by Voigt functions with fixed Gaussian contribution to the spectral broadening especially estimated for the spectrometer prior to the measurement.

## 4. Conclusions

In conclusion, Raman-active, mode-sensitive spectroelectrochemical point measurements with single-digit µm resolution of the charge transfer reaction were compared with the standard Tafel plot approach of integral electrochemistry involving the response from the whole cathode. The presented approach has shown the potential of mechanistic insight into the changes occurring at the cathode material γ-$Li_{x}V_{2}O_{5}$. The vibrational insight into the electrochemical changes at the cathode material with Raman spectroscopy allowed us to follow the polymorphic phase transformation pathway of the lithium vanadium pentoxide at the cathode. A large dissonance was found in the anodic oxidation and cathodic reduction proportionality factors α. Based on detailed spectral analysis at the varying potentials, a mechanistic pathway of lithium migration and restoration of crystalline $V_{2}O_{5}$ from the highly lithiated $Li_{x}V_{2}O_{5}$ is proposed. Raman shifts of the investigated Raman-active modes of lithium vanadium pentoxide were found to follow the current development at the cathode. On the other hand, Raman intensity followed the trends only for selected modes, which may indicate a preferred symmetric pathway of $Li^{+}$ migration. In addition, the possibility of using the response of the Raman shift instead of the current readout was presented. The Raman shift was found to follow the trend of the electrochemical measurement. Due to the response of the Raman signal to the applied potential, the current readout in the Tafel plot was replaced by the Raman shift, and striking similarities of charge transfer reaction coefficients were found. Similar conclusions could not have been drawn from only the integral standard electrochemical measurements. On the other hand, when only the Raman intensity was used without compensation for fluorescence effects, the results were not quantifiable. In terms of outlook, the vibrational-structure-resolved point spectroelectrochemistry may open new possibilities to implement online measurements of the battery performance in operando modus. It may allow the detection of changes occurring within the material with much higher precision than by integral and pure electrochemical methods.

There is a possibility of increasing the detection limit by the application of surface-enhanced Raman spectroscopy (SERS), for example, by using gold nanoparticles (SHINERS), which is, nevertheless, outside the scope of this article. Due to the spectral character of the Raman electrochemical analysis, the limiting factor being laser spot and Raman cross section, it provides much more precise insight into the cathode composition, as compared with pure electrochemical methodology, with its integral character.

## Figures and Tables

**Table 1:** Comparison of laser readout and current readout in Tafel plot analysis and kinetic merits. The explanation of the presence of the $B_{1u}$ mode in the Raman spectrum is provided in the text.

Raman Mode ${}^{a}$	Chemical Bond	$\alpha_{\mathrm{cathodic}}$	$\alpha_{\mathrm{anodic}}$
$A_{g}$ ${}^{b}$	V=O(1)	0.88	0.12
$B_{1g}$ ${}^{c}$	V-O(2)	0.90	0.10
$B_{1u}$ ${}^{d}$	V-O	0.92	0.08
$B_{2g}$ ^e^	V-O(3)	0.80	0.20
$B_{3g}$ ${}^{f}$	V-O(4)	0.87	0.13
$\Sigma_{Average}$		0.87	0.13
E-chem		0.84	0.16

${}^{a}$ g = gerade (Raman active), u = ungerade (see text); ${}^{b}$ Mode at 951 cm${}^{-1}$; ${}^{c}$ Mode at 901 cm${}^{-1}$; ${}^{d}$ Mode at 842 cm${}^{-1}$; ^e^ Mode at 642 cm${}^{-1}$; ${}^{f}$ Mode at 540 cm${}^{-1}$.

## Supplementary Materials

The following are available online, Figures S1–10, Measurement protocol and Requirements for the measurement.
