# Supplementary material for: Operando Raman Shift Replaces Current in Electrochemical Analysis of Li-ion Batteries: A Comparative Study
Source: Molecules. 2021 Aug 1;26(15):4667. doi: 10.3390/molecules26154667 (PMC8439359; doi:10.3390/molecules26154667)
Supplement: Supplementary file 1 [file molecules-26-04667-s001.zip › molecules-1318505-supplementary.pdf]

Operando Raman shift replaces current in Butler-Volmer analysis of Li-ion batteries: a comparative study
Supporting information

M. Radtke[a], C. Hess
Technical University of Darmstadt
Eduard-Zintl-Institute of Inorganic and Physical Chemistry
Alarich-Weiss-Str. 8
64287 Darmstadt, Germany
[a] corresponding author: mariusz.radtke@tu-darmstadt.de

July 31, 2021

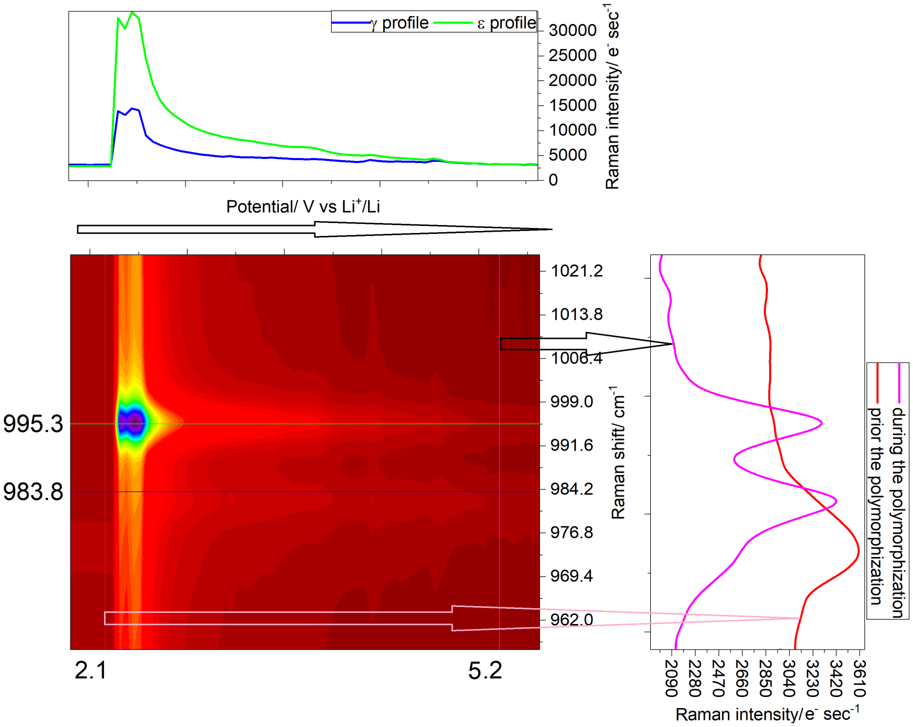

Figure S 1: Operando polymorphization measurement of the γ to ε-$Li_xV_2O_5$ transitions as a cause of the electrochemical scan, as indicated by the distinctive blue-shift of over 40 cm^{-1} from 954 to 995 cm^{-1} of the A$_g$ mode.

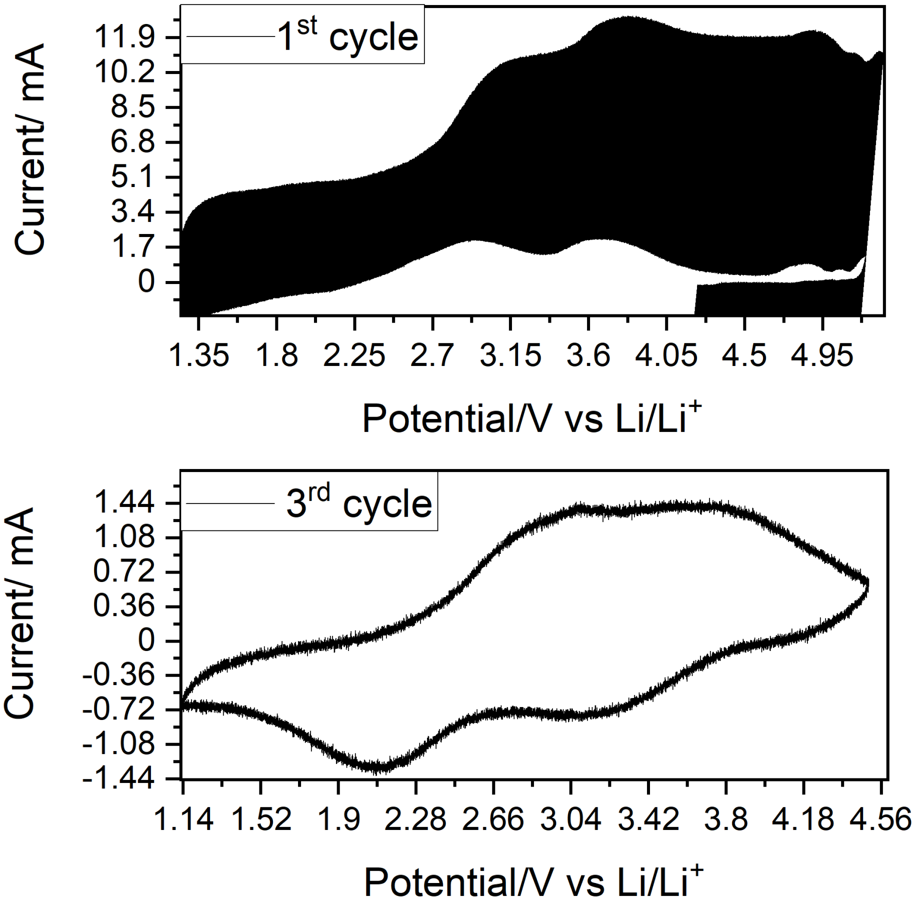

Figure S 2: Differential Pulse Voltammetry (DPV) scan of γ-$Li_xV_2O_5$ in the first and second cycle showing the influence of omitting the incorporation of the binder into the cathode composition and the effect of relatively large potential window reaching 5.2 V vs Li/Li^+. A non-reversible decomposition of the electrolyte and possibly a generation of meta-stable vanadyl complex with ethylene carbonate (EC) is shown on example of the first cycle. By reducing the potential window on the same battery in the following measurement shows no considerable influence of the SEI-layer on the electrochemical performance.

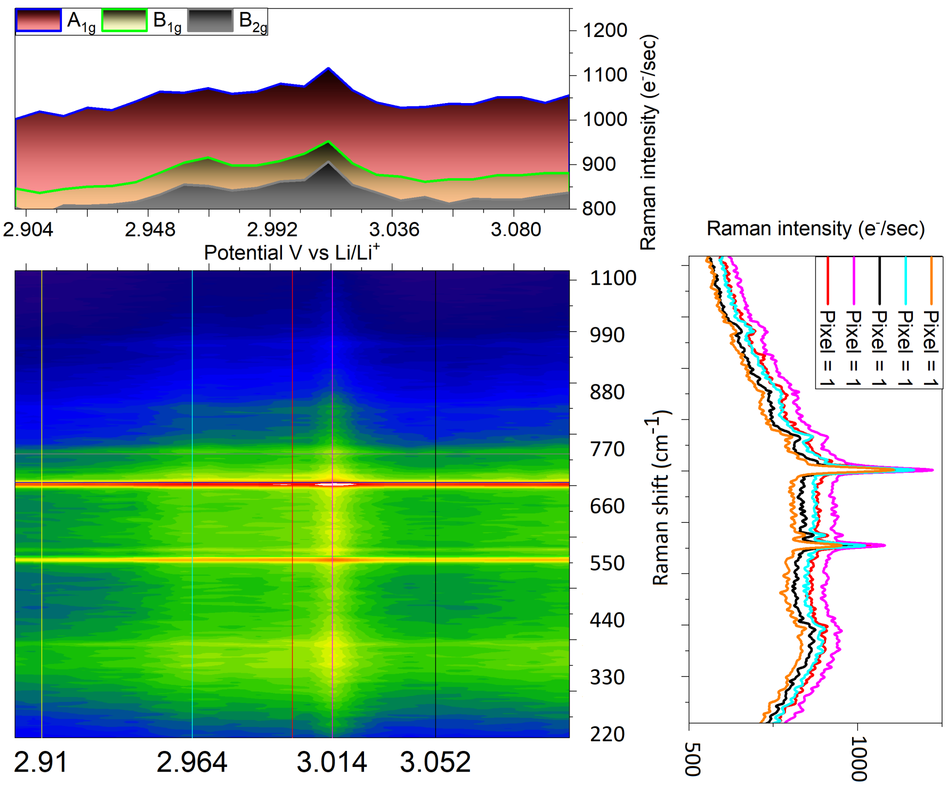

Figure S 3: Example of time resolved Raman specta of $Li_xV_2O_5$ over the measurement time. Three Raman active modes were selected and are shown in the upper layer. The layer to the right shows the arbitrarily chosen Raman spectra prior electrochemical change, during electrochemical change and after it. The in-text mentioned mirroring when only Raman intensity (e^-/sec) is used is clearly visible. The middle layer represents the changes in Raman intensity (coloured z-scale) as a function of the applied potential. The Raman intensity can be attributed to each active mode.

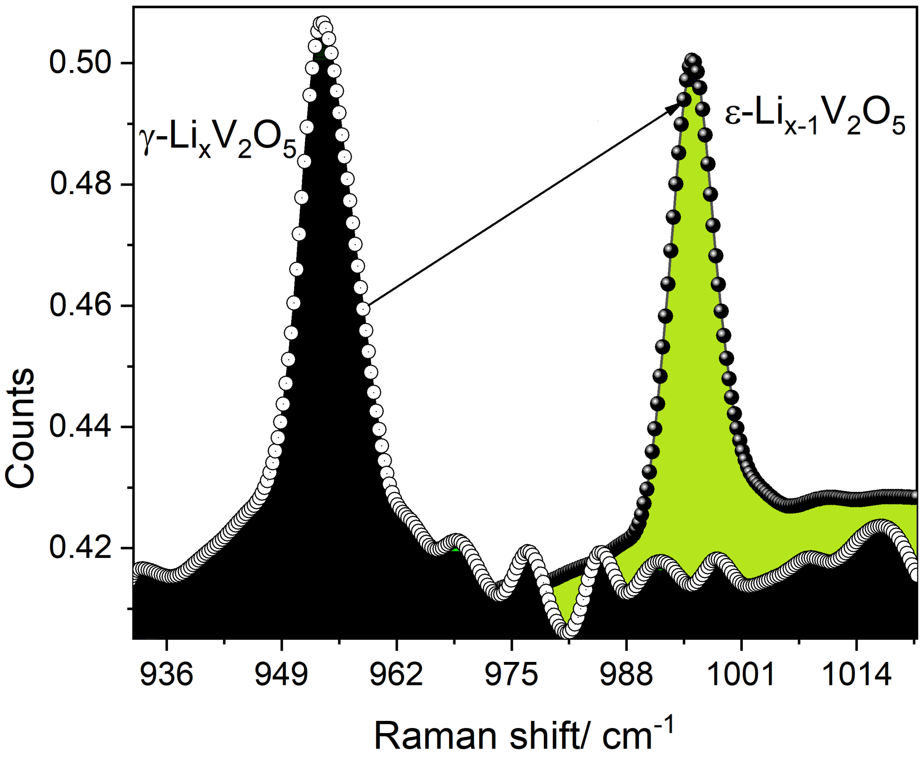

Figure S 4: Detailed look at the initial and final stages of the $Li_xV_2O_5$ polymorphization from γ to ε.

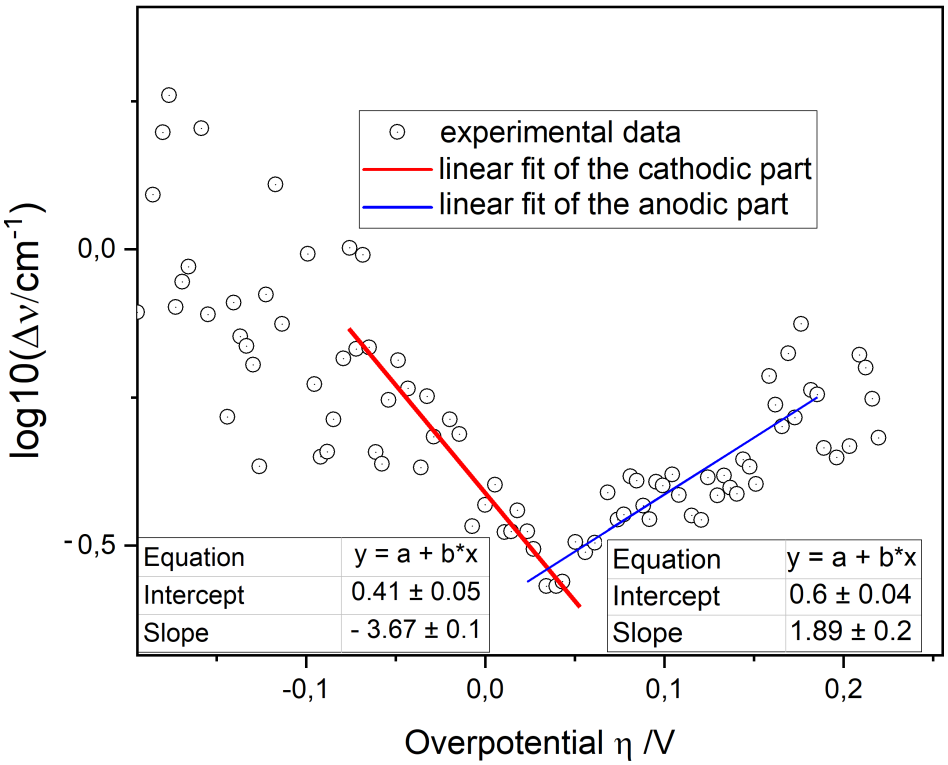

Figure S 5: Example of calculation of the heterogeneous charge transfer proportionality factor according to Butler-Volmer and respective anodic and cathodic parts of the Tafel plot.

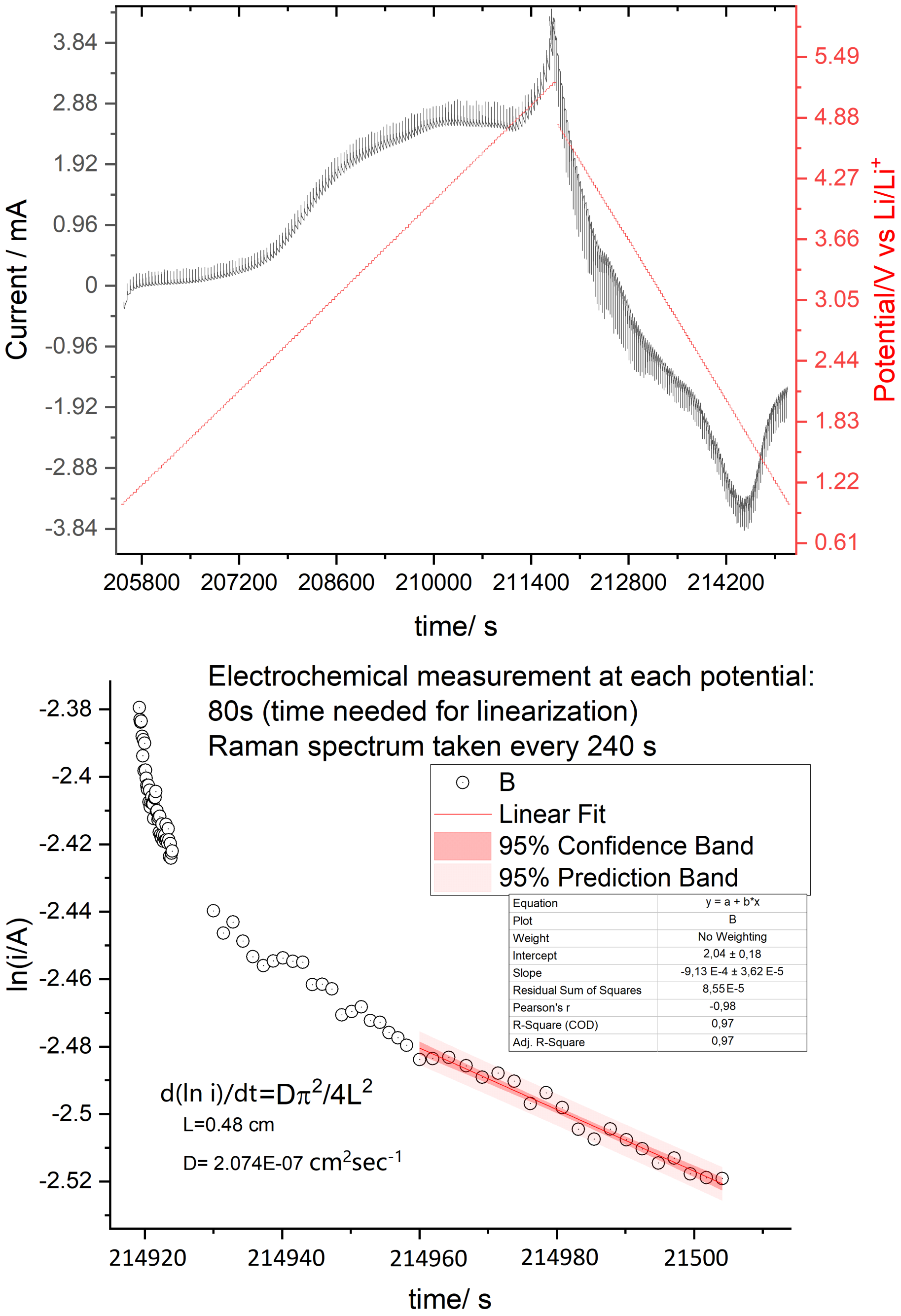

Figure S 6: Potentiostatic Intermittent Titration Technique (PITT) of the $\gamma - Li_xV_2O_5$ with the calculation of the time needed for the linearization of the current, as judged by the presence of stable diffusion coefficient and linear region of the current relaxation.

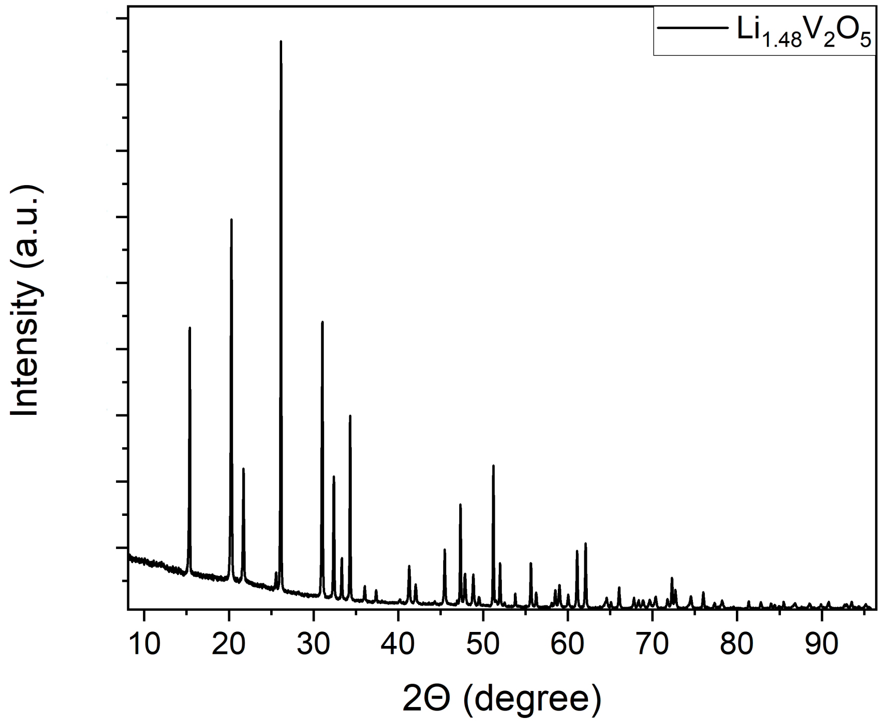

Figure S 7: Powder XRD data of the synthesized $Li_xV_2O_5$ sample.

Sample	x	O at%	V at%	Li at%	Li/V	V/O
LixV2O5	1.48	17.20	6.80	5.03	1.48	1:2.5

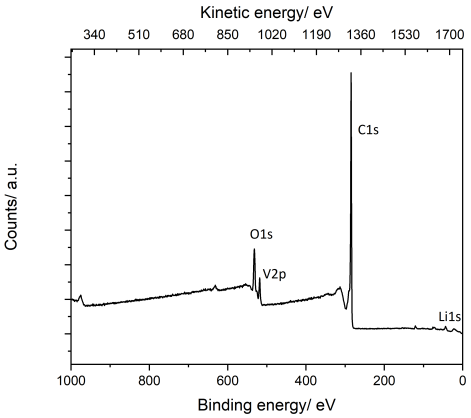

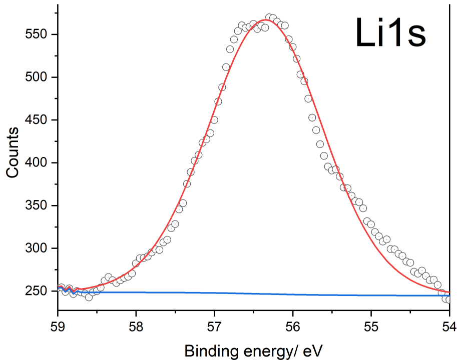

Figure S 8: XPS data of the synthesized $Li_xV_2O_5$, the "x" amount was elucidated from the narrow scan analysis.

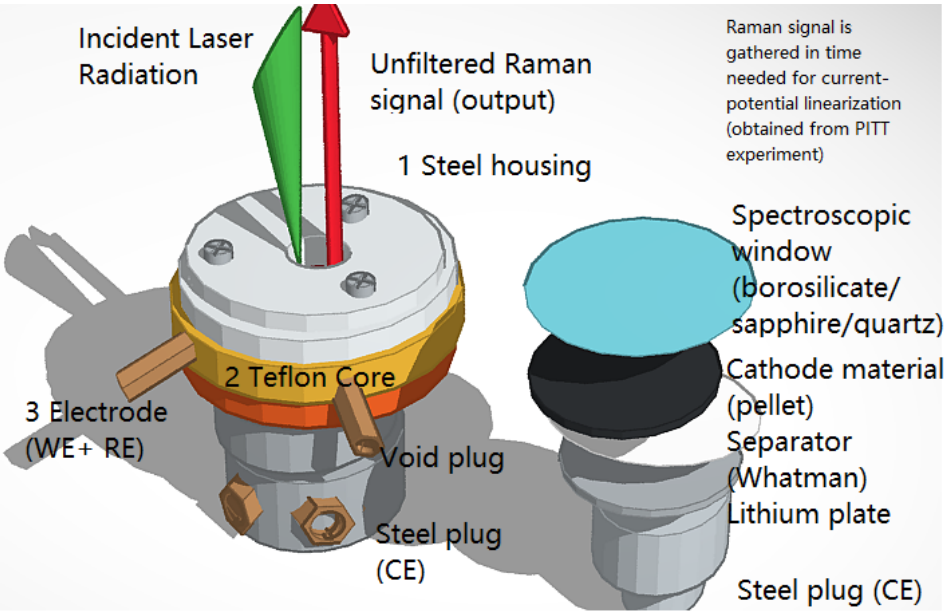

Figure S 9: Raman spectroelectrochemical cell (EC-Lab GmbH, Hamburg, Germany) with pellet system allowing for spectroelectrochemical analysis with minimum amount of electrolyte signals.

1 Requirements for the measurement

The requirements for the measurement require investigation of material‘s Raman cross-section in ex-situ modus, while special attention needs to be paid for elimination of the electrolyte signals by applying the assembly procedure described above. The long-range min. 50x microscope objectives with high numerical aperture are suggested for performing the analysis. The knowledge of crystallinity of the material is required from powder XRD in order to estimate the capability of Raman measurement.

2 Measurement protocol

Electrochemical cell assembly:
In order to perform spectroelectrochemical measurement of Butler-Volmer/Tafel analysis, the following procedure was employed. The cell assembly was performed under the inert atmosphere of argon with O_2 and H_2O contents below 0.5 ppm. The schematic drawing of the cell is depicted in the Figure 9. For visible range lasers borosilicate glass was used, while we note that for smaller wavelenghts (and higher irradiation energies) quartz and sapphire glass (up to

250 nm) are more suitable (due to the transmittance reasons). The teflon-core of the cell was closed with a stainless steel plug from the bottom (Figure 9), which here was functioning as counter-electrode (CE). The thin ring punched out of lithium foil was placed on the stainless steel-plug, which was then covered with the Whatman filter paper, serving as a separator. The separator was soaked with the minimum amount of electrolyte, which allowed for the conductivity without dilution of the possibly diffusing electrode material. The pellet of cathode material was placed on top of the filter paper and was covered with borosilicate glass.

Raman spectroelectrochemical measurement:

The potential range was applied to the cell in a range covering the electrochemical activity. Raman spectrum was acquired in time periods of current linearization, which may be obtained from PITT experiment (Figure 6). Series of Raman spectra were evaluated by a standardized procedure involving background correction and fitting with Voigt functions, while merits of Raman shift, FWHM and intensity were extracted from each spectrum. The merits were then related to the Raman acquisition time. The acquired time was then related to the overpotential during the polarization measurement. By plotting the decadic logarithm of the Raman shift against overpotential, the spectroelectrochemical-Tafel-plot was generated, which was the starting point for further analysis. An exemplary z-scan for the depth penetration is shown in the Figure 10, as based on a (400) single crystal CVD-grown diamond commonly used as a standard for laser intensity callibration in our setups.

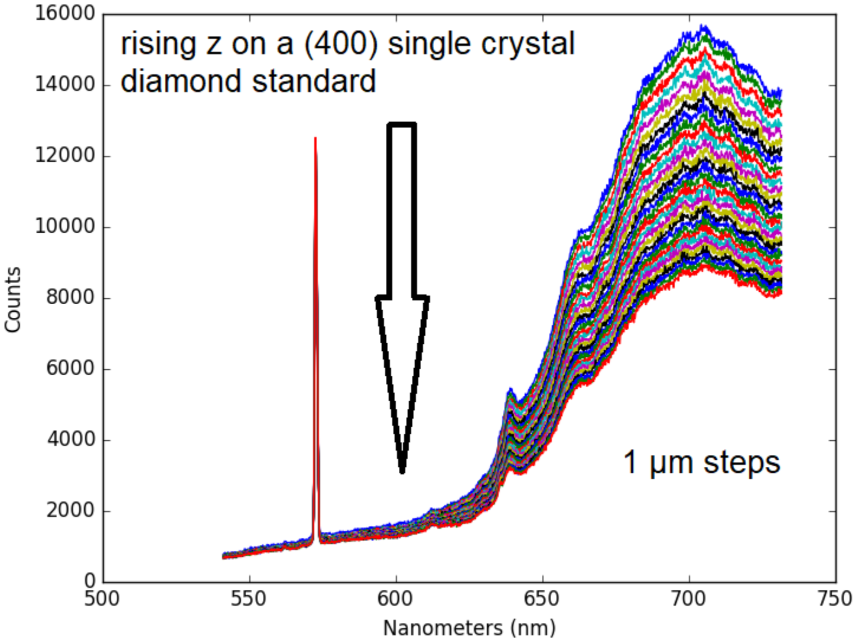

Figure S 10: Representative z-scan for the estimation of the depth penetration needed prior each measurement in order to reach the Raman focus for materials with different cross-sections.
